# Clinical Symptomatology of Anxiety and Family Function in Adolescents—The Self-Esteem Mediator

**DOI:** 10.3390/children11030338

**Published:** 2024-03-12

**Authors:** Ignatia Farmakopoulou, Maria Lekka, Evgenia Gkintoni

**Affiliations:** 1 Department of Education and Social Work, University of Patras, 26504 Patras, Greece; ifarmakop@upatras.gr; 2 Department of Medicine, University of Athens, 11527 Athens, Greece; mlekka@med.uoa.gr; 3 Department of Psychiatry, University General Hospital of Patras, 26504 Patras, Greece

**Keywords:** clinical psychology, anxiety, systemic therapy, family functioning, adolescence, cohesion, adaptability, self-esteem

## Abstract

Background: Family, the child’s first environment, shapes their psycho-emotional balance. The literature links adolescent anxiety to family relationships, interactions, and dynamics. The self-esteem of adolescents appears to protect their mental health. Goal: This study examines whether family cohesion and adaptability affect adolescent anxiety symptoms. It also examines whether teen self-esteem mediates this relationship. Method: This cross-sectional, descriptive study included 166 Attica youth aged 12–18 from schools and educational units. The adolescents completed Olson’s FACES-III cohesion and adaptability scale, Spielberger’s STAI-C, Rosenberg’s self-esteem scale, and a socio-demographic questionnaire. Results: Family cohesion, but not adaptability, was negatively correlated with state (*rho* = −0.25, *p* = 0.001) and trait (*rho* = −0.46, *p* < 0.001) anxiety in the adolescents. Teenagers from extreme families with the lowest cohesion and adaptability had higher trait anxiety (*x*^2^(2) = 6.91, *p* = 0.032) than those from moderately balanced/balanced families. Self-esteem mediated the relationship between the family cohesion functioning and adolescent’s state anxiety (*p* = 0.005) and trait anxiety (*p* = 0.011). Conclusions: The findings show that family dysfunction negatively impacts adolescent anxiety, as well as their self-esteem, which protects mental balance.

## 1. Introduction

### Concept, Roles, and Forms of Family

The family serves as the primary and fundamental social environment in which a child undergoes development and acquires the fundamental aspects of their personality. From a theoretical perspective, a family can be defined as a group of individuals who cohabit in a shared dwelling, bound together by familial connections through blood relations, marital unions, or legal adoption, who rely on one another for various forms of support encompassing physical, emotional, material, and financial aspects. A theorist of family therapy posits that the family, as a crucial social institution, can be conceptualized as a “system of subjectivities that engage in continuous interactions.” Conversely, alternative perspectives proposed by theorists and researchers of family therapy contend that the family functions as a psychosocial system characterized by its internal dynamics, roles, rules, and member interactions.

According to research [1], the family’s primary and essential function is to fulfill its members’ functional, physical, and emotional requirements. The family is responsible for meeting essential needs, including the supply of sustenance, housing, and material possessions, while safeguarding its members from external threats and offering social support [2,3]. Simultaneously, the interconnectedness among family members, the potential for personal growth and self-identification, the encouragement of individual creativity and initiatives, and the facilitation of social integration collectively fulfill crucial functions within the family unit, addressing the mental and emotional well-being of its constituents [4,5,6,7].

The theoretical framework surrounding the family as a system emerged in the post-World War II era, when psychoanalytic therapists Nathan Ackerman and Murray Bowen observed, through their therapeutic work, that the difficulties experienced by the patients were intricately linked to the dynamics of their familial relationships [8,9,10]. From a systemic viewpoint, there has been a change in focus from the individual to the family setting in which their conduct occurs, as well as the interactions between family members [11]. The reciprocal influence of the system members, the interrelationships among them, the delineation of system borders, and the presence of individual subsystems collectively contribute to the formation of human behavior. The systemic view acknowledges that an individual’s dysfunctional conduct indicates dysfunction within the familial system that they are a part of.

The study of the family system from a systemic perspective has led scholars to examine the various aspects and processes that contribute to its functionality or dysfunctionality. The researchers who adopt a systemic view examine various patterns related to the functioning of the family system, including the interactive sequences among family members, the cohesive ties that exist between them, the clarity of the roles within the family, and the ability to adapt to stressful conditions [11].

Based on the examination of multiple theoretical models, it is evident that the critical features of a well-functioning family include the presence of solid interpersonal bonds among its members, the provision of emotional support and accessibility from parents to their children, effective communication channels, and the ability to adapt roles and rules during challenging circumstances [1].

The scope of the present research study is to investigate the relationship between the family environment, specifically cohesion and adaptability, and the presence of anxiety symptoms in adolescents. Simultaneously, it examines whether adolescents’ self-esteem is a mediator in this relationship.

## 2. Literature Review

### 2.1. Self-Esteem Factor

The cohesive functioning of families’ and adolescents’ psychological well-being, particularly regarding anxiety symptoms, appears to be influenced by self-esteem. According to family therapy theorists [12], self-esteem is an individual’s view of themselves and their attitude toward themselves. Self-esteem is an individual’s positive or negative self-evaluation. High self-esteem is a sign of a person’s self-worth and is linked to life satisfaction and mental health. Self-esteem differs from self-concept, which is a person’s view of themselves. In contrast, self-esteem is linked to an individual’s perception of their abilities and qualities, which are part of the self [13].

From a lifespan perspective, preschoolers have high self-esteem. This phenomenon is limited during school. Children can self-evaluate more accurately as their cognitive faculties mature and social comparisons with peers emerge [14]. Biological changes, like puberty and emotional development, and social changes, like identity and personality formation, mark adolescence. Self-esteem catalyzes the adolescent’s experience of these changes [15].

In the early stages of this developmental era, self-esteem appears to be limited due to social comparisons with peers, limited educator attention, and profound changes. However, during middle adolescence, self-esteem rises due to increased autonomy, the sense of control over one’s life, and the expansion of social networks and participation in activities that develop individuality [16,17,18,19]. Research links high self-esteem in adolescence to better academic performance [20]. Self-esteem also improves social support and physical and mental health [13]. Teens with low self-esteem are more likely to develop internalizing disorders, substance abuse, and suicidal tendencies [21].

According to empirical evidence, females, especially adolescents, have lower self-esteem than males. According to a study [13], girls and boys develop self-esteem differently due to their interpersonal interactions and personal achievements. Girls obtain their self-worth from relationships, while boys obtain it from their family, social, and academic performance. In addition, peer and social relationships are essential for self-esteem in adolescence. Using 961 adolescents, a study [21] examined self-esteem and other social factors. The study found that teens with high self-esteem had more social support, more extensive social networks, and friendly interactions. In addition, self-esteem protects teens from delinquency, aggression, and academic underachievement.

Several studies have shown that interpersonal relationships and interactions throughout an individual’s life shape their self-esteem [22]. However, family and parent–child relationships are crucial. Bowlby’s attachment theory states that a newborn’s secure attachment to its mother during infancy promotes emotional security, acceptance, and love. Secure attachment promotes self-esteem and value. More specifically, parental emotional availability and warmth help children develop mental representations of themselves as worthy of care and affection [23].

Individuals develop a strong sense of self-worth that lasts throughout their development. Research [5] suggests that children raised by accessible parents who respect their children’s autonomy, acceptance, and active communication have higher self-esteem. In contrast, authoritarian parents with limited emotional availability and responsiveness and frequent reprimands appear to harm adolescents’ self-perception. This can cause insecurity, weakness, and inadequacy [24].

Many studies support the above conclusions, including a recent longitudinal study [25,26,27,28]. Their study of 500 Spanish adolescents found a strong link between parenting styles, self-esteem, and peer relationships. Democratic parenting improves peer relationships, self-esteem, self-efficacy, and academic performance. In addition, two studies on an adolescent cohort found that “abusive” parenting practices—authoritarianism, parental rejection, strict control, and disciplinary measures—are linked to school bullying, marginalization, and lower self-esteem [29,30]. Using physical violence as a disciplinary measure for teens has been linked to adverse outcomes like lower self-esteem, increased aggression, and poor interpersonal relationships.

In contrast, parents who interfere with their adolescent children’s freedom and autonomy, especially when they are actively seeking independence, appear to lower their self-esteem [30]. Promoting teenage autonomy from parental figures, cultivating initiative-taking behaviors, actively engaging in family decision making, and establishing flexible, democratic, and transparent family limits are associated with higher self-esteem. Another study [31] found that adolescents who view their parents’ communication as positive, supportive, and open are more likely to have positive self-images than those who view it as overly controlling.

In the end, distant parents show indifference and emotional distance toward their children. Although children are given opportunities to develop their autonomy and individuality, emotional presence during their development needs to be improved, and they are not guided when their attempts at autonomy fail. Ambivalent parenting is also linked to adolescent self-esteem and powerlessness [32].

### 2.2. Family Function and Self-Esteem

Previous research has examined the relationship between functional aspects of the family system, like coherence and adaptability (Olson’s Circumplex model), and children’s self-esteem. Another study [32] found that family cohesion and parental warmth affect adolescent self-esteem. As mentioned above, the study emphasizes family adaptability, including the ability to set flexible boundaries, change rules, and involve adolescents in decision making. These factors help teens develop positive self-images.

Some researchers [33] examined family cohesion and adaptability, family system functionality, and self-esteem in 18–22-year-olds. Strong family bonds, effective communication, and siblings have been linked to higher self-esteem. However, there is little evidence that adaptability is similar. Researchers [33] found that adolescents with low self-esteem came from dysfunctional families. These adolescents had anxiety, depression, and antisocial behavior.

A recent study [34] examined 316 delinquent 12–18-year-olds. They found that an impaired family environment, characterized by low cohesion and adaptability, hinders adolescents’ emotional and physical growth. Therefore, this environment fosters feelings of insecurity and parental neglect, which are linked to lower self-esteem and self-perception in adolescents.

Another study [35] involved 1040 adolescents and their parents. This study found a link between parental depression and family dysfunction. Teens with this dysfunction may have low self-esteem. Additionally, dysfunction and low self-esteem increase the risk of adult psychopathology. Depression and anxiety symptoms were also examined in the same study [35].

A recent longitudinal study [30] examined how the family environment affects self-esteem in late childhood and adolescence. A sample of 674 children and their families was studied. According to the same researchers [30], parental warmth, positive family relations, harmonious parent–child relationships, father involvement in upbringing, and lower depressive symptoms in the mother positively correlate with adolescent self-esteem.

### 2.3. Family Functionality and Anxiety

Family structure has a significant impact on adolescents’ mental health, particularly their anxiety symptoms. This literature review gathers papers with similar findings.

A study of 691 14–19-year-olds found that adolescents from dysfunctional families, characterized by parental alcohol use, low familial cohesion, ineffective communication, and ongoing marital discord, had extroversion difficulties. Aggression, impulsivity, and substance abuse were issues. These individuals also experienced introversion-related issues like depression, anxiety, and suicidal tendencies [36]. Researchers [37] examined the relationship between family structure, functionality, and psychological well-being. No matter the family structure, a functional family system with adaptability, member cooperation, mutual emotional support, and practical problem solving protects mental health, according to the researchers.

The dysfunctional family environments in which adolescents are raised and their introversion challenges are linked. Another systematic study [30] linked dysfunctional homes, parental psychopathology, and adolescent anxiety and depression. As mentioned above, the review found that introversion-related challenges in teens can predict family conflict and harm parents’ mental health.

In their meta-analysis [38], researchers examined the relationship between teen anxiety symptoms and parental traits. A lack of warmth, acceptance, and availability toward adolescents, parental rejection, and strict control were found to be significant predictors of adolescent anxiety symptoms. To be more specific, parental rejection impairs adolescents’ emotional regulation. This increases their susceptibility to worry, increasing their risk of anxiety symptoms. According to the same study [38], strict parental supervision and encouraging adolescents to depend on their parents when they are ready to function independently reduces personal competence and increases anxiety.

Researchers also conducted a randomized controlled clinical experiment [39] on 104 anxiety-diagnosed adolescents and 44 community-selected adolescents. The experimental group of anxiety-disordered teens showed more family dysfunction, less adolescent autonomy, and lower family cohesion than the control group. However, the differences between the cohorts were not statistically significant. Cognitive–behavioral therapy improved the group cohesiveness, self-governance, and anxiety symptoms in the experimental cohort.

A community sample of 1395 adolescents and their families was studied longitudinally. This study found that psychopathology in the parents and an unfavorable family environment with restricted interpersonal bonds, excessive protectiveness, or the rejection of the adolescents significantly increase the likelihood of anxiety symptoms in the adolescents. This population was especially prone to social anxiety disorder [40]. A previous study on 174 adolescents, averaging 16 years old, found that a lack of independence, an excessive sense of familial unity marked by fusion, and parents’ tendency to shield their adolescent offspring excessively were associated with increased adolescent stress levels. Research [41] also linked parental arguments to adolescent nervous symptoms and family triangulation.

A recent study [42] examined marital subsystem conflicts in homeless adolescents. The research suggested that early childhood trauma, dysfunctional familial relationships, increased parent–child conflict, and parent–adolescent conflict were linked to introversion-related issues like anxiety and depression. The study also linked these traits to adolescent extroversion issues like crime and substance use.

In a significant study [43], the McMaster model examined family functioning and its many characteristics. This study examined the relationship between dysfunctional family systems and anxiety symptoms in young adults with predisposition anxiety. The study found a link between anxiety and low-income family functioning. Individuals predisposed to anxiety had a stronger association [43]. A cohort of young adults with anxiety disorders was studied to determine the family system functioning [44]. According to the study, the families had negative traits like low emotional engagement and communication. Additionally, the individuals’ quality of life was significantly impacted.

Researchers found a link between suboptimal parenting and reduced family adaptability and cohesion in a prominent longitudinal study of 11–16-year-olds from low-income and single-parent households. This study found several predictors of anxiety and depression in adolescents who experienced chronic and daily stressors throughout their lives. A study [45] found that stress negatively impacts the family system, making teens more susceptible to anxiety and depression symptoms.

### 2.4. Family Functionality in the Level of Cohesion and Adaptability, Adolescent Anxiety, and Self-Esteem

Research has consistently shown that family system attributes affect mental health. Family dysfunction can cause anxiety in adolescents. This association is not linear or straightforward. Critical factors like self-esteem may influence the phenomenon [9]. The literature suggests that a dysfunctional family environment with limited cohesion, poor communication, rigidity, and inflexibility causes adolescents to feel vulnerable and insecure, compromising their self-esteem [35,46]. The study [33] found that a low self-image increases the risk of psychopathologies like anxiety.

Family dysfunction may be linked to anxious and depressive symptoms in adolescents with physical illnesses, according to a longitudinal study [47]. The study included 10,646 kids and teens. The study found that stress from physical illness is linked to maternal depression and family disruption. Therefore, these factors lower children’s self-esteem, increase insecurity, and cause anxiety and depression symptoms.

Parent–child conflicts often involve verbal aggression, according to a study on children’s self-esteem, parental hostility toward their children, and family cohesion and anxiety symptoms. Consequently, children develop low self-esteem and self-perception, which increases anxiety [48]. This study supports the idea that a cohesive, nurturing, and practical family setting reduces the risk of anxiety in teens by providing emotional support and fostering a positive self-image [49].

A study [8] examined how self-esteem and school-related stress affect the relationship between familial dysfunction and adolescent anxiety. The results from their research were significant. The study found a link between familial-context disruption and teen anxiety. Self-esteem also moderated this association. School stress was a key moderator in the same study [8]. In a previous study [50], self-esteem mediated. Conditional parental acceptance, parental unavailability, strict parental control, and intrusive parenting strategies, which disrupted family harmony, were linked to lower adolescent self-esteem. The same researchers [50] found a link between low self-esteem and anxiety and depression symptoms.

A separate cognitive–behavioral study in adolescents examined how self-esteem and loneliness affect the relationship between dysfunctional families and depression and anxiety. Family dysfunction, as measured by Epstein’s McMaster model, was linked to teens’ negative self-image and relationships with parents and peers. This caused low self-esteem and loneliness. Skewed perceptions caused anxiety and depression, which affected their mental health [51].

The manifestation of anxious symptoms in teens, precisely social anxiety symptoms, was also studied. This study examined the relationship between social anxiety symptoms, adverse life events, family functioning, and adolescent self-esteem. This study shows that domestic violence, parental substance use, and marital problems cause family dysfunction and lower adolescent self-esteem. Low self-esteem is linked to anxiety, particularly social anxiety disorder symptoms [51].

The evidence shows that self-esteem significantly affects family dysfunction and teen anxiety symptoms. According to [52], an organized home environment boosts self-esteem, which protects teens’ mental health. A recent family-based study on self-esteem, hope, family cohesion, and adolescent mental health found significant results. According to [52], family cohesion and close relationships can protect adolescents’ mental health. This may be because it boosts self-esteem.

### 2.5. Research Goals and Hypotheses

The previous evaluation of the scholarly literature demonstrates the correlation between family cohesion adaptability and stress levels in adolescents, particularly emphasizing the mediating role of self-esteem. Due to the low amount of research conducted on the synergistic effects of the factors being investigated, the primary objective of this study is to contribute to the current body of literature by examining the link between the variables in question. The study also possesses practical significance by emphasizing the protective role of self-esteem in safeguarding the psychological well-being of adolescents residing in dysfunctional home settings.

The main research aims were as follows:

- [RQ1] To examine the correlation between family functionality and its dimensions, namely, cohesion and adaptation, and anxiety symptoms, namely, predisposition and state anxiety, among adolescents.
- [RQ2] To determine variations in stress levels, self-esteem, and demographic factors related to socioeconomic contexts and family structures across different types of families within the total sample. These types include extreme families characterized by low levels of cohesion–adaptability, moderately balanced/balanced families, and families at an intermediate level;
- [RQ3] To precisely identify the potential mediating role of self-esteem in the link between the key variables.

The research hypotheses/questions of the study were formulated as follows:

The presence of dysfunction in the cohesion and adaptability of the family environment is considered a risk factor for developing anxiety symptoms, including both state and predisposition anxiety, in adolescents;

Adolescents residing in highly dysfunctional family contexts are more prone to experience heightened levels of anxiety in comparison to their counterparts in moderately balanced family environments;

Adolescents hailing from families characterized by high levels of functioning are more inclined to originate from a socioeconomic stratum of affluence and possess a nuclear family configuration, in contrast to their counterparts belonging to families of the moderate or extreme types, which exhibit lower levels of coherence and adaptability;

It is anticipated that girls exhibit higher levels of anxious symptomatology compared to boys, and that adolescents aged 15–18 have higher levels of anxious symptomatology compared to those aged 13–15.

Adolescents originating from low socioeconomic backgrounds and non-nuclear family structures are anticipated to exhibit comparable levels of elevated stress compared to their counterparts from medium and upper socioeconomic backgrounds and families with uncertain structures;

The anticipated level of cohesion and adaptability within the family is expected to be lower among adolescents from low socioeconomic backgrounds than those from middle and high socioeconomic backgrounds. Similarly, adolescents from families with non-nuclear structures are expected to exhibit lower levels of cohesion and adaptability compared to those with unclear family structures.

The association between family functioning, specifically cohesion and adaptability, and the stress experienced by teenagers is influenced by the mediating factor of their self-esteem.

## 3. Materials and Methods

### 3.1. Planning

The present research is a cross-sectional, descriptive study that was carried out at the School of Medicine of the University of Athens. The study took place in school units and a tutoring unit of second-grade education of the Attica region. The data were collected over three months, from December 2021 to March 2022, while the overall study lasted until June 2022.

### 3.2. Participants

The study sample was a community one, specifically including teenagers aged 12–18 years from secondary-school units, as well as from a tutoring unit, in the municipality of Attica in Greece.

The survey used randomized sampling, and the total sample was 166 adolescents from four different schools. Specifically, the total sample consisted of N = 81 boys (48.8%) and N = 85 girls (51.2%).

### 3.3. Inclusion/Exclusion Criteria

Adolescents aged 12–18 could participate in the study; they had to speak and understand the Greek language and have the written consent of their parents for their participation. At the same time, oral consent was obtained from the adolescents for their participation. As an exclusion criterion, the existence of a diagnosed disorder on the spectrum of autism, mental retardation, or a diagnosed psychiatric disorder was set, for the reason that they would not be able to complete the questionnaires of the study and the completion could possibly be burdensome for their mental balance.

### 3.4. Procedure

As part of the study’s conduct process, following the drafting of the research protocol, a letter was written to the principals of the second-grade school units of the Attica region. The letter included information about the study’s aims, its content, and the details of the researcher and the study supervisor, and it was signed by the director of the postgraduate program and the researcher. In the second year, this letter was sent electronically to the addresses of secondary schools and tutoring educational units in various areas within Attica. The units to which the letter was sent were 15 in total; the selection was coincidental and, of these 15 units, 5 responded positively.

Subsequently, for the units from which we received a positive response, telephone contact was initially made with the managers, and a first face-to-face meeting was arranged with the researcher to inform them in detail about the study, its aims, the participants, and the tools. At this meeting, they were given the study questionnaires, as well as the letter in paper form, as the consent form to the parents. After fully informing them, the classes in which the researcher would administer several questionnaires to the teenagers were decided based on the inclusion and exclusion criteria of the study.

Then, the director of each unit sent the written consent form in electronic form to the parents of the teenagers who belonged to the classes for which the administration of the questionnaires had been pre-decided. At the same time, specific dates and times were defined for each unit to which the researcher would visit the classes to administer the questionnaires to the teenagers. The parents who were sent the written consent forms of their children in the study had to send them electronically by the date of the researcher’s second visit.

On the designated dates, the second visit of the researcher was made to the units, where the management initially assured her of the consent of the parents of the teenagers for their participation in the study. Then, the researcher visited the classes that had been selected. Before the lesson started, the questionnaires to be filled in were given to the teenagers who met the participation criteria. At the same time, the adolescents were informed that the completion was optional and anonymous and would take 15–20 min. The researcher was present for any questions. In some classes, the teachers were equally present in the process. Regarding the adolescent participation rate, no adolescents presented a refusal from themselves or their parents to participate in the study.

### 3.5. Data Collection Tools

#### 3.5.1. Family Adaptability and Cohesion Evaluation Scale—FACES-III

The FACES-III was designed by the authors of [53] and was based on the theoretical background of the “Combination Model of Marriage and Family Systems” (Circumplex Model of Marital and Family Systems). This version is the third revised version of the questionnaire. It is a self-report questionnaire, consisting of 20 questions, and assesses family functioning through two central dimensions: cohesion and adaptability [53]. Based on a theoretical framework, there is also a third dimension of communication, which, however, plays an auxiliary role and is not measured by the FACES questionnaire.

Higher values in the dimension of cohesion and in the dimension of adaptability, respectively, indicate balance and more orderly functioning in the family system [53]. Responses are given on a five-point Almost Never–Almost Always scale. All family members can complete the scale. In the present study, it was completed by the teenagers.

In terms of the cohesion dimension, it is measured by the odd-numbered questions, and the individual concepts evaluated are emotional attachment (11.19), family boundaries (7.5), support and alliances (1.17), time and friends (9.3), and leisure interests (13.15). Also, the dimension of cohesion has four different escalating levels based on the rating: distanced, separated, connected, and highly connected families. In terms of the dimension of adaptability, it is measured by the questions with even numbers, and the individual concepts evaluated are leadership (6.18), control (2.12), discipline (4.10), and roles and rules set within the family (8, 14, 16, 20). Based on the scoring, the family can be characterized as “rigid”, with the lowest levels of adaptability, or as “structured”, “flexible,” and “very flexible,” with the last level representing the maximum adaptability.

Through the questionnaire and the combinations resulting from the levels of cohesion and adaptability, three types of families are formed: extreme families (Extreme types), those at the intermediate level (Mid-Range), and those characterized by balance (Balanced types) [53]. The scale presented satisfactory conceptual construct validity and good internal consistency reliability with Cronbach’s indexes of 0.97 for the coherence dimension and 0.96 for adaptability.

In the present study, the Cronbach’s reliability index for the dimension of coherence was found to be 0.85, and that for adaptability was 0.79, indicating acceptable scale reliability.

#### 3.5.2. State-Trait Anxiety Inventory for Children (STAI-C)

The STAI-C questionnaire was constructed by the researchers of [54]; it is a self-report questionnaire and consists of two individual subscale dimensions: state anxiety (A-State) and predisposition anxiety (A-Trait). The state anxiety dimension includes 20 questions and assesses how the subject feels, examining situational anxiety. Higher values indicate higher levels of situational anxiety. Answers are given on a three-point scale through which the subject states the intensity of their feelings at the time of administration (for example, “I feel very calm”, “calm”, and “not at all calm”). Questions 1, 3, 6, 8, 10, 12, 13, 14, 17, and 20 indicate the absence of anxiety and are reverse-scored, while the rest indicate the presence of anxiety. The total score can range from 20 (minimum anxiety value) to 60 (maximum anxiety value).

The anxiety predisposition dimension also includes 20 questions and examines anxiety as a personality trait (i.e., whether the subject tends to experience anxiety in general throughout their life). Higher values indicate that the subject is more prone to perceive various social situations as threatening and to react with anxiety. Responses are given on a three-point scale, “Rarely–Very Often”, depending on the frequency of the occurrence of the behavior described. All questions indicate the presence of anxiety, and the score ranges from 20 to 60.

Moreover, in the state anxiety dimension, the factor Absence of Anxiety showed high internal consistency reliability (Cronbach’s a = 0.85), and the factor Presence of Anxiety showed a = 0.83. In the predisposition anxiety dimension, the internal consistency reliability was equally high (Cronbach’s a = 0.85), while the dimensions also showed good levels of conceptual construct validity [54]. In the present study, the internal consistency reliability (Cronbach’s a) in the dimension of state anxiety was found to be 0.81, and in that of predisposition anxiety, it was found to be 0.76.

#### 3.5.3. Rosenberg Self-Esteem Scale

Rosenberg’s self-esteem scale was created in 1965 by Rosenberg and is a tool for evaluating an individual’s self-esteem (i.e., their sense of personal worth). It is a tool that has been translated and adapted in many countries, with widespread use in general and clinical populations. It consists of 10 questions–statements to which the subject is asked to answer on a four-point scale: “Strongly Agree–Strongly Disagree” [12].

Statements 1, 3, 4, 7, and 10 are worded positively (for example, “I have a positive attitude towards myself,”), while statements 2, 5, 6, 8, and 9 are worded negatively (“Sometimes I think I’m no good at all”) and they reverse the grading. A higher score indicates the higher level of self-esteem of the subject. In the present study, the researcher found the Cronbach’s a index to be 0.77.

#### 3.5.4. Socio-Demographic Questionnaire

In order to collect the social and demographic data of the participants, the demographic data questionnaire was constructed, which included questions regarding the gender, age, and class of the adolescent participants, their areas of residence, the existence of siblings, their family situation (whether the parents were together/married or separated), and their family structure (whether the teenagers lived with both parents, with only one parent, with parents and relatives, or with one parent and his/her partner).

In addition, the questionnaire included questions about the professional status and educational level of the parents, as well as the socioeconomic context of the family. Finally, it was investigated whether, in the past, the adolescent participants had visited a mental health specialist. The adolescents completed the questionnaire along with the other psychometric tools provided.

### 3.6. Statistical Analysis

The statistical package SPSS 22.0 was used to analyze the data collected in the research. Initially, the distributions of the quantitative variables were checked for the normalities of their distributions with the Kolmogorov–Smirnov test. For those that were normally distributed, mean values and standard deviations were used to describe them, while for those that were not normally distributed, medians and interquartile ranges were additionally used. Absolute (N) and relative (%) frequencies were used for the qualitative variables. The non-parametric Mann–Whitney test was used to compare the quantitative variables between two groups. In comparison, the non-parametric Kruskal–Wallis test was used to compare the quantitative variables of more than two groups. At the same time, to check for type I errors due to multiple comparisons, the Bonferroni correction was used, based on which the significance level was 0.05/κ (κ = the number of comparisons).

Also, the Spearman correlation coefficient (rho) was used to check the relationship between the two quantitative variables. In order to find the factors independently associated with anxiety and to test the mediating role of self-esteem in the relationship of the FACES with anxiety, a multivariate hierarchical linear regression was performed from which the dependence coefficients (β) and their standard errors (SEs) were derived. Initially, the demographic and family elements and the cohesion dimension were entered into the model with successive inclusions–removals. At the second level, the self-esteem scale was introduced.

To test for mediation, the Sobel test that is mentioned in [55] was employed, which is based on the product of the coefficients a and b and is known as the multiplication of the coefficients. In the present study, the independent variable must first be significantly related to the dependent variable of the study or the family functioning from the level of coherence and adaptability to stress. Second, the independent variable must be related to the mediating variable, the self-esteem factor. Third, the mediating variable must be correlated with the dependent variable. Furthermore, after introducing the mediating factor, the effect of the independent variable on the dependent one is reduced. In this case, there is partial mediation; if it becomes non-significant, there is total mediation.

With these conditions, the mediation of self-esteem in the relationship of family functioning at the level of cohesion and adaptability with the stress scale was tested. Linear regression analysis was performed using logarithmic transformations. Internal reliability was tested using Cronbach’s a. Significance levels were two-sided, and statistical significance was set at 0.05.

## 4. Results

The study sample comprised 166 individuals enrolled in high school or who had recently graduated. The sample consisted of adolescent pupils categorized into three categories based on their homes’ cohesion and adaptability levels. These groups were explicitly classified as extreme, medium, and somewhat balanced/balanced households. Thirty-seven teenagers, accounting for 22.3% of the sample, were categorized as belonging to extreme families. Additionally, 83 adolescents, representing 50% of the sample, were classified as belonging to medium families, while 46 adolescents, making up 27.7% of the sample, were categorized as belonging to somewhat balanced/balanced families. The demographic data on the entire sample and categorized by family type are displayed in Table 1.

Within the entirety of the sample, it was observed that 81 participants, accounting for 48.8% of the total, were identified as males, while 85 individuals, constituting 51.2% of the total, were identified as females. The sample consisted of 50 individuals between the ages of 13 and 15, representing 30.1% of the total sample. Additionally, there were 116 participants between the ages of 15 and 18, accounting for 69.9% of the sample. Most participants (31.3%) were enrolled at the 1st Lyceum. Concerning familial circumstances, it was found that 71.1% of the participants had parents who were married, 7.8% had parents who were separated, and 21.1% had divorced parents. Furthermore, it is worth noting that most participants, precisely 95.2%, reported that both parents were alive. In contrast, a smaller proportion, namely, 4.8%, indicated that they had experienced the unfortunate event of losing one of their parents.

Concerning the categorization of family structures, it was found that 61.4% of the adolescent participants originated from a nuclear family structure. In contrast, the remaining 38.6% were derived from alternative family structures, including single-parent, reconstituted, and extended families.

Concerning the presence of siblings, it was found that 77.1% of the adolescent participants reported having brothers. Concerning their parents’ occupational statuses, 35.5% indicated that their fathers worked as private employees, while 33.7% reported that their mothers had private employee positions. Furthermore, it is worth noting that 38 students, representing 22.9% of the sample, were identified as coming from poor socioeconomic backgrounds. In contrast, most students, specifically 113 individuals, or 68.1%, were classified as belonging to middle socioeconomic backgrounds. Lastly, a smaller proportion of the participants, namely, 15 youngsters, or 9%, came from high socioeconomic backgrounds. Concerning the parents’ educational backgrounds, it was found that 53.6% of the student population had fathers who had obtained higher education degrees. In comparison, 63.9% had mothers who had achieved the same level of educational attainment.

Upon conducting a comparative analysis of the elements within the table, it was observed that various factors, namely, the marital status (χ^2^(4) = 11.45, *p* = 0.017), family structure (χ^2^(6) = 14.85, *p* = 0.015), socioeconomic background (χ^2^(4) = 29.53, *p* < 0.001), father’s educational level (χ^2^(4) = 10.05, *p* = 0.027), and mother’s educational level (χ^2^(4) = 10.17, *p* = 0.017), exhibited statistically significant differences across the different family types. In moderately balanced/balanced families, there was a more considerable prevalence of parents who were together or married, a higher proportion of nuclear families, a more significant occurrence of high socioeconomic environments, and a higher percentage of fathers and mothers with higher education levels [RQ1].

Table 2 presents the scores obtained by the adolescent participants concerning the aspects of the FACES-III cohesion–adaptability scale, STAI-C anxiety scale, and self-esteem scale. Greater values indicate increased degrees of cohesion and adaptability, heightened anxiety, and enhanced self-esteem. The coherence dimension exhibited a range of scores from 12 to 50 points, with a median value of 34 points (range: 28–40 points). The scores of the adaptability dimension exhibited a range of 12–47 points, with a median value of 25 points (range: 22–30 points).

Furthermore, the scores obtained on the anxiety predisposition scale exhibited a range of 23–71 points, with a median value of 35 points (range: 32–41 points). The state anxiety scale yielded scores ranging from 25 to 60 points, with a median value of 40 points (individual range: 35–45 points). The self-esteem scale yielded scores ranging from 15 to 39 points, with a median score of 26 points (range: from 23 to 29 points).

Significant differences were observed in the cohesion dimension (χ^2^(2) = 80.40, *p* < 0.001), the adaptability dimension (χ^2^(2) = 84.86, *p* < 0.001), and on the predisposition anxiety scale (χ^2^(2) = 6.91. *p* = 0.032) when comparing the scales across the different family types. Adolescents who were members of households characterized by moderate balance or balance had considerably greater cohesion and adaptability than those from homes with extreme or medium levels of balance. Following the implementation of the Bonferroni correction, it was observed that adolescents originating from extreme family environments had notably elevated levels of predispositional anxiety compared to their counterparts from moderately balanced family backgrounds [RQ1].

The cohesion and adaptability components of the FACES are presented in Table 3, providing the individual levels for the overall sample and by gender. A significant proportion of the adolescent population, precisely 50.6%, exhibited the lowest degree of cohesion within the context of distant families. Conversely, a notable percentage of teens, precisely 27.7%, had the highest level of adaptability within the framework of very flexible homes. There was no statistically significant difference in the levels of coherence and adaptability between boys and girls (*p* > 0.05). Concerning family typologies, it was found that 22.3% of the teenagers were affiliated with extreme families. In comparison, 50% were associated with medium families, and the other 27.7% were categorized as belonging to balanced or moderately balanced households. There was no significant difference in family type between boys and girls (*p* > 0.05) [RQ1].

### 4.1. Correlations between Survey Scales

Concerning the associations among the key variables examined in the research, as depicted in Table 4, a statistically significant positive connection was observed between cohesiveness and the self-esteem scale, with a correlation coefficient of 0.24 (*p* = 0.002). Additionally, a substantial positive correlation was found between cohesion and adaptability, with a correlation coefficient of 0.22 (*p* = 0.005). Furthermore, there was a notable positive correlation between predisposition anxiety and state anxiety, with a correlation coefficient of 0.60 (*p* < 0.001). There was a substantial negative correlation between predisposition anxiety and the self-esteem scale (rho = −0.42, *p* < 0.001), as well as between predisposition anxiety and coherence (rho = −0.46, *p* < 0.001). In a similar vein, it was shown that state anxiety exhibited a substantial negative correlation with both the self-esteem scale (rho = −0.63, *p* < 0.001) and coherence (rho = −0.25, *p* = 0.001). There was no observed correlation between the adaptability dimension of the FACES and the two anxiety dimensions measured by the STAI-C or self-esteem scales [RQ2, RQ3].

### 4.2. Correlation of Anxiety with Student Demographic Characteristics

The data shown in Table 5 illustrate the scores of the students on the predispositional anxiety and state anxiety scales, categorized by their demographic features. Concerning gender, a notable disparity in the anxiety tendency was observed, with girls exhibiting considerably higher levels (U = 2553.5, *p* = 0.004) than boys. Concerning age, no statistically significant disparities were detected in the susceptibility to anxiety between individuals aged 13–15 and those aged 15–18. Concerning the state anxiety dimension, a statistically significant disparity was detected based on gender, wherein females exhibited higher values than boys (U = 1780.5, *p* < 0.001). Simultaneously, it was observed that teenagers aged 13–15 exhibited higher levels than those aged 15–18, with a U statistic of 2185 and a *p*-value of 0.012. Simultaneously, there was no discernible variation in the levels of anxiety experienced by adolescents about their family’s marital status, parental status, family structure (nuclear or non-nuclear), or the presence of siblings [RQ2].

In addition, concerning the group of adolescents, there was a statistically significant difference in the prevalence of anxiety (χ^2^(5) = 12.38, *p* = 0.030), with the highest rates observed among third-year high school students. Concerning the socioeconomic status of the adolescent population, there was a notable variation in the tendency toward anxiety scores across different levels. This difference was found to be statistically significant, as indicated by the chi-square test (χ^2^(2)= 6.24, *p* = 0.044). Following the implementation of the Bonferroni correction, the results revealed a statistically significant difference in the anxiety levels between students of low socioeconomic status and teenagers from middle socioeconomic backgrounds (*p* = 0.014). This finding suggests that students with lower socioeconomic status experience higher levels of worry. A significant variation was found in the state anxiety dimension, contingent upon the socioeconomic backgrounds of the adolescents (χ^2^(2) = 7.34, *p* = 0.025). Following the implementation of the Bonferroni correction, the study’s results indicated that adolescents hailing from poor socioeconomic backgrounds exhibited significantly elevated levels of state anxiety compared to their counterparts from middle socioeconomic backgrounds (*p* = 0.008) [RQ2].

### 4.3. Correlation of Cohesion–Adaptability Family Functioning with Students’ Demographic Characteristics

Table 6 presents the scores of the adolescent participants in the domains of family functioning, specifically cohesion and adaptability, categorized by their demographic features. In the dimension of coherence, there was a notable disparity in the scores of the adolescents’ parents’ marital statuses based on whether their parents were married or divorced. Students with married parents exhibited significantly higher scores than those with divorced parents (U = 1616.5, *p* < 0.001). Furthermore, it was observed that families characterized by a nuclear structure exhibited significantly better levels of cohesion than families with alternative structures (U = 2244.5, *p* = 0.01).

Moreover, there was a significant variation in the coherence dimension scores across different student classes (χ^2^(5) = 11.19, *p* = 0.048). Notably, the students from the first high school had the highest scores. The results indicate a significant difference in the scores for the coherence dimension based on the socioeconomic setting (χ^2^(2) = 27.62, *p* < 0.001). Following the application of the Bonferroni correction, it was determined that, in instances in which the context was low, the score exhibited a statistically significant decrease in comparison to situations in which the context was medium (*p* < 0.001) or high (*p* = 0.001).

In the facet of adaptability, there was a statistically significant difference in the adaptability scores between pupils whose parents were living (U = 373, *p* = 0.050). A statistically significant difference was also found concerning the socioeconomic level (χ^2^(2) = 20.53, *p* < 0.001). The Bonferroni correction was conducted post hoc and revealed that the score was considerably lower at a low level compared to both the medium level (*p* < 0.001) and high level (*p* < 0.001). The results indicate a significant difference in the adaptability scores based on the educational level of both the fathers (χ^2^(2) = 6.38, *p* = 0.041) and mothers (χ^2^(2) = 8.39, *p* = 0.015). Individuals with parents who had higher education had the highest adaptability scores [RQ2].

### 4.4. Self-Esteem as a Mediating Factor in the Relationship between Family Functioning and Stress

The following conditions must be met to investigate the potential mediation of self-esteem in the association between family functioning and stress. The FACES (cohesion and adaptability) exhibits a notable correlation with the dependent variable of anxiety as measured by the STAI-C scale. Moreover, it is worth noting that the FACES exhibits a noteworthy association with self-esteem. Furthermore, self-esteem correlates with anxiety as measured by the STAI-C scale. Based on the current study, it is evident that among the dimensions of the FACES, only the dimension of “cohesion” demonstrated a significant link with the dimensions of “situation anxiety” and “predisposition anxiety” as measured by the STAI-C scale. Simultaneously, there was a notable correlation between cohesion and self-esteem. Ultimately, there was shown to be a significant link between self-esteem and both categories of anxiety. Therefore, given that the three essential criteria were satisfied, a mediation analysis was conducted to examine the role of self-esteem in the association between the coherence dimension and both predispositional anxiety and situational anxiety. The findings are presented in the tables provided below.

It is worth mentioning that no significant correlation was observed between the “adaptability” dimension of the FACES cohesion and adaptability scale and the dependent variable of anxiety across the dimensions. Consequently, a mediation analysis was not conducted, as it would have required meeting the essential condition of a correlation between the independent and dependent variables.

The researchers conducted a multivariate hierarchical linear regression analysis to investigate the potential mediating role of self-esteem in the association between cohesiveness and predisposed anxiety. The results of this analysis are presented in Table 7 and Table 8. During the initial phase (step 1) of the study, the model incorporated demographic and family factors and the cohesion dimension. These variables were introduced into the model using the sequential-inclusion–removal method. Next, in phase 2, the self-esteem scale was implemented.

The initial stage of the study revealed a significant independent relationship between gender and the coherence component concerning predisposed anxiety. Upon introducing the self-esteem measure into the model, the impact of gender diminished, although the cohesion factor remained significant. Therefore, a substantial correlation was observed between dispositional anxiety and coherence and the self-esteem scores [RQ3].

This study revealed that self-esteem has a mediating function in the association between cohesiveness and predisposed anxiety. The mediation was deemed partial, as the significance of the connection persisted even after accounting for self-esteem in the model. The results of Sobel’s test indicate a statistically significant level of partial mediation (*p* = 0.011).

The researchers employed a comparable methodology to examine the role of self-esteem as a mediator in the association between cohesion and the state anxiety component. The multivariate hierarchical linear regression analysis involved the initial inclusion of demographic and familial factors and the cohesiveness dimension using the sequential-inclusion–removal approach. Subsequently, self-esteem was incorporated into the concept.

According to the data shown in Table 9 and Table 10, the variables of gender, age, and the coherence dimension exhibited independent associations with state anxiety during step 1 of the analysis. All the variables mentioned above retained their statistical significance upon the inclusion of the self-esteem measure in the model. The dimension of cohesiveness, despite its limited association with the dependent variable, remained statistically significant. Hence, the relationship between coherence and state anxiety was partially mediated by self-esteem. The Sobel test yielded a statistically significant result (*p* = 0.005), indicating the presence of partial mediation.

## 5. Discussion

The main focus of this study was to examine the correlation between family functioning, specifically in terms of cohesion and adaptability, and the manifestation of anxiety symptoms, including state anxiety and predispositional anxiety, in adolescents. Consequently, the study sought to investigate whether their self-esteem played a mediating role in this relationship. The primary objective was to enhance the existing literature on the phenomena above, as well as to investigate the mental well-being of adolescents residing in dysfunctional family settings.

The statistical analysis of the study indicates that the dysfunction of the family environment is a risk factor, to some degree, for the development of anxiety in teenagers. Specifically, while there was no statistically significant connection between the adaptability dimension and stress, the cohesion dimension of the FACES-III cohesion–adaptability scale showed a negative correlation with both dimensions of stress. This confirms that higher levels of family cohesion are associated with lower levels of dispositional anxiety and state anxiety in adolescents.

Simultaneously, it was discovered that adolescents originating from highly dysfunctional families exhibited significantly higher levels of anxiety in the predispositional anxiety dimension in comparison to adolescents from moderately balanced families. Additionally, these adolescents displayed lower cohesion and adaptability within their families. These findings partially validate the initial and secondary research hypotheses of this study.

According to multiple studies, including the recent research conducted by the researchers in [26], strong family bonds, emotional support, and accessible parents are linked to reduced stress levels in teenagers. Simultaneously, a noteworthy discovery from the study revealed that family cohesion is a protective factor against the manifestation of anxiety symptoms in early adulthood [26].

Teenagers raised in environments lacking emotional warmth and parental availability exhibit greater emotional instability when confronted with stressful events, leading to heightened anxiety [56]. Adolescents’ perception of their parents as accessible, generous, and supportive, particularly during challenging times, aids in the cultivation of their self-efficacy and capacity to manage negative emotions in the face of stressful situations [57].

Moreover, a familial setting marked by persistent conflicts between parents and adolescents and between parents, as well as a lack of unity, induce emotional insecurity and unfavorable perceptions of familial dynamics in adolescents. These circumstances frequently result in various adjustment difficulties, including anxiety. The adolescent’s emotional security is jeopardized by a threat that triggers fear and anxiety, prompting them to seek security. If this persists, it can result in the development of anxiety symptoms [58].

According to a study [59], enhancing family cohesion during adolescence can decrease introversion issues by boosting self-esteem. Multiple studies have demonstrated that when families have stronger bonds, adolescents benefit from improved problem-solving abilities, enhanced emotional control, and reduced interpersonal challenges. These factors collectively contribute to shielding them from developing anxiety and other mental health issues [28,60,61,62].

Furthermore, the commencement of adolescence appears to be correlated with issues in the unity and overall operation of the family, which contribute to the emergence of anxiety and other difficulties in adolescents. According to a study [63], the transition into adolescence brings about several changes in children, such as the need to establish their identity, emotional instability, and physical transformations. These changes appear to disrupt the functioning of the family, resulting in imbalances, frequent conflicts with parents, and a growing distance between teenagers and parents. These alterations restrict the efficient operation of the family and strain adolescents’ psychological well-being [63].

Furthermore, aside from the changes that occur during adolescence, the presence of stressful factors is also associated with disruptions in the functioning of the family environment and, consequently, contributes to the increased stress experienced by adolescents. According to a study conducted by researchers in [64], being exposed to chronic daily stressors and non-stress factors is linked to decreased functioning within the family, which, in turn, leads to the development of anxious symptoms in adolescents.

Conversely, an excessive level of cohesion among family members contributes to the dysfunction of the family structure. The heightened emotional intimacy between parents and teenagers frequently results in excessive safeguarding and the erosion of the teenager’s inherent desire for self-governance and self-reliance. The interaction between the catalyst and the developmental demands of adolescence, which necessitate the readjustment of the family system equilibrium, exposes adolescents to the possibility of experiencing anxiety and other symptoms of psychopathology. The excessive protection and involvement of parents in the lives of teenagers gradually diminish the teenager’s sense of autonomy, resulting in a sense of powerlessness and the emergence of anxiety [7].

According to a study conducted by researchers in [53], there is a curvilinear relationship between cohesion and family functioning. This means that both a lack of cohesion and excessive cohesion can lead to dysfunction in the family environment. This relationship is mainly observed in clinical populations, in which families may have deficient or excessively high levels of cohesion. The latter can result in a lack of autonomy for adolescents. This relationship between cohesion and the functionality of the family framework has not been confirmed in non-clinical populations. Higher levels of cohesion in the family system result in more organized functioning and the better protection of the mental well-being of its members. This parallel relationship is also demonstrated in the current study’s findings, which were obtained from a population with no clinical conditions.

A noteworthy discovery from the study is that the adaptability dimension of the FACES coherence and adaptability scale did not exhibit a statistically significant correlation with either the state anxiety dimension or the predispositional anxiety dimension. The level of adaptability within the family system, which refers to its capacity to modify dynamics, boundaries, and rules in response to new circumstances, did not appear to impact the development of anxiety symptoms in adolescents. This finding is consistent with the Greek study conducted by researchers [65], which utilized the cohesion and adaptability questionnaire to examine family dynamics. The study revealed that accurately describing the functioning of Greek families and assessing potential variations in areas such as rules, member roles, and discipline proved challenging when considering the adaptability dimension.

Regarding the third research hypothesis, it was anticipated that adolescents belonging to different types of families (extreme, middle, and moderately balanced/balanced) characterized by varying levels of cohesion and adaptability would exhibit differences in their socioeconomic backgrounds and family structures. Specifically, it was expected that a higher percentage of adolescents from the moderately balanced/balanced family type would come from high socioeconomic backgrounds and have a nuclear family structure.

The results indicate that adolescents from moderately balanced/balanced families had higher rates of the nuclear family structure and came from higher socioeconomic backgrounds compared to the other family types. This finding is statistically significant. In moderately balanced or balanced families, there was a higher proportion of parents who were married or together, as well as a higher percentage of parents with higher levels of education.

According to the study conducted by researchers in [66], families that exhibited superior internal functioning were found to have a nuclear family structure, as opposed to other types of family structures. In addition, families from low socioeconomic backgrounds frequently experience a range of internal issues, including intra-family disputes, child neglect, limited communication among members, and an inability to address various problems effectively. These elements impact their functionality [66].

In this study, we examined whether there are variations in the expression of anxiety symptoms (both current and predisposed) among adolescents concerning different demographic factors. The results indicate that girls exhibited significantly higher levels of state anxiety and predispositional anxiety compared to boys, as hypothesized.

Multiple studies have consistently shown that adolescent girls are more susceptible to experiencing symptoms of anxiety compared to boys [5,67]. This phenomenon can be attributed to various factors, including the hormonal fluctuations that occur during puberty, the heightened cognitive processes that reinforce anxiety, and the greater likelihood of girls, as compared to boys, to report their symptoms of anxiety [68]. Furthermore, research has shown that girls are more likely than boys to internalize their anxiety, while boys are more prone to exhibit extroversion-related difficulties during this developmental stage [36].

Moreover, the heightened occurrence of girls being exposed to instances of abuse, particularly sexual abuse, during their childhood and adolescence is linked to higher rates of anxiety and coexisting disorders. Studies on situational and dispositional anxiety have found that girls are more susceptible to experiencing both persistent and prolonged anxiety during adolescence, as well as intermittent anxiety, because they tend to react with anxiety to different life situations [28].

Studies indicate that anxiety levels tend to rise in adolescents during late adolescence, as reported by researchers [36]. The current study found that adolescents aged 15–18 exhibited a higher average level of predispositional anxiety compared to younger individuals. Nevertheless, this discrepancy was not deemed statistically significant.

Adolescents aged 13–15 exhibited a statistically significant increase in anxiety in the state anxiety dimension when compared to adolescents aged 15–18. This finding indicates that the adolescents’ involvement in the current study served as a source of stress for the younger adolescents. As a result, they exhibited a higher stress level in response to the situation after the study was completed. Furthermore, the study questionnaires were administered in the initial months of the school year, when the teenagers, particularly those who had recently started secondary education, had yet to fully adjust. Consequently, their emotional well-being may have been affected by their involvement in the study. Simultaneously, as corroborated in the research conducted by researchers in [69], the combined impact of the substantial developmental changes that adolescents must navigate during the early stages of adolescence is what contributes to their elevated levels of anxiety in comparison to subsequent years of age [69].

Regarding the socioeconomic context, the study found a connection with anxiety. Specifically, teenagers from low socioeconomic backgrounds showed the highest levels of state anxiety and a greater predisposition compared to those from medium and high socioeconomic backgrounds. This supports the original hypothesis of the study.

Extensive research has consistently shown that adolescents who grow up in low socioeconomic environments, characterized by poverty, long-term financial struggles within their families, and a lack of adequate social support, are more likely to experience anxiety, as well as other issues related to introversion and extroversion [70,71]. Adolescents from low socioeconomic backgrounds may experience a diminished sense of self-confidence and heightened anxiety due to the financial strain on their families. These factors contribute to the development of anxiety symptoms. Furthermore, a study conducted by Wadsworth and colleagues revealed that a family’s low socioeconomic status is a contributing factor to the adoption of authoritarian parenting practices in child rearing. This, in turn, results in the development of stress and puts a general strain on the mental well-being of the children [72].

Concerning the family structure, it was anticipated that teenagers from nuclear families would exhibit lower levels of stress in comparison to those from alternative structures, such as single-parent, reconstituted, or extended families. According to a recent study [73], teenagers who were raised by single parents or in blended families experienced elevated levels of stress compared to those from traditional nuclear families, irrespective of their gender.

Adolescents in single-parent families following divorce experience heightened mental health challenges, primarily stemming from the stressful circumstances associated with divorce. These include frequent parental conflicts and the financial hardships that the family must navigate [74].

Nevertheless, the current study’s findings indicate that adolescents from nuclear families do not exhibit statistically significant variations in their anxiety levels, in either state anxiety or predispositional anxiety, when compared to those from families with different structures. Despite contradicting the initial hypothesis, this finding is supported by multiple studies, including the research conducted by the authors of [37]. Their cross-sectional study demonstrated that the family structure, as opposed to family functionality, had no impact on the mental health of the participants.

Simultaneously, a parallel investigation conducted on a group of adolescents revealed that the configuration of their families did not serve as a prognostic indicator for the introversion issues they encountered. However, the emotional attachment and unity within their families, as perceived by the teenagers themselves, impacted their introversion problems [75]. According to a study [76], nuclear families are more effective than single parents at safeguarding the mental well-being of teenagers, provided that the family is functioning adequately. Nevertheless, there are instances in which family dysfunction arises, leading to a complicated relationship and making it difficult to observe the impact of the family structure on the mental well-being of the adolescent members [76].

Therefore, it seems that stress in teenagers is more closely linked to the instability of the parental role and the frequent conflicts between parents, particularly in single-parent families. These factors contribute to teenagers experiencing anxiety and other mental health issues [77].

The study examined the relationship between demographic factors and the cohesion and adaptability of families. The results showed that teenagers from low socioeconomic backgrounds had lower cohesion and adaptability levels than those from medium and high socioeconomic backgrounds. This finding was in line with the research hypothesis and was statistically significant.

Undoubtedly, based on the existing literature, families residing in low socioeconomic backgrounds, who are subjected to poverty, high unemployment rates, and social isolation, reside in deteriorated regions with elevated crime rates and face difficulties in fulfilling the necessities of their children. Families frequently encounter internal challenges, including conflicts among family members, the neglect of children, insufficient communication, and difficulty resolving various issues, all impacting their overall functioning.

According to the Family Stress Model, families in low socioeconomic contexts experience high stress levels due to financial burdens. This stress negatively affects the relationship between the parents and children, leading to problems in the family’s functioning and impacting the mental health of the children. Furthermore, researchers [56] conducted a recent study positing that economic hardships experienced by low-income families substantially impact the parent–adolescent relationship. These difficulties lead to heightened conflicts and diminished cohesion, as parents feel incapable of fulfilling the developmental requirements of their adolescent children [56].

Furthermore, the findings revealed that families with a nuclear structure exhibited significantly higher levels of cohesion than those with different structures. The study [66] provides evidence that the nuclear family structure is linked to superior family functioning compared to alternative structures. In addition, the authors of [78] conducted a comparable study. They contended that single parenthood is linked to decreased organization within the family and places a strain on the mental well-being of the child members.

In contrast, researchers [9] found that parental conflicts and tensions negatively impact the family climate, parent–child relationships, and children’s satisfaction with the family functioning. This effect was observed in both nuclear and single-parent families, and it had significant implications for the mental health of adolescents aged 12–16. These ambiguous results highlight the need for additional research examining the correlation between family structure and functioning.

Notable discoveries were made regarding the correlation between demographic factors and family functioning regarding cohesion and adaptability. Specifically, it was found that teenagers whose parents were married exhibited significantly higher levels of cohesion. In comparison, those whose parents were alive and had higher education demonstrated significantly higher levels of adaptability.

The final research hypothesis posited that self-esteem would mediate the relationship between family functioning, specifically cohesion and adaptability, and stress. Statistical analyses confirmed that self-esteem partially mediates the relationship mentioned above. It was discovered that there is a direct correlation between the level of cohesion within a family and adolescents’ self-esteem. In other words, when there is a strong bond and unity within the family, the adolescent is more likely to have higher self-esteem and to experience less stress, both in terms of their predisposition and current situation.

To summarize, self-esteem appears to play a protective role in the development of anxiety in adolescents. When self-esteem is considered, the impact of the family-context cohesion on adolescent anxiety is restricted. Notably, there was no statistically significant relationship between the adaptability dimension of the FACES and stress. Therefore, the issue of self-esteem mediating the relationship between adaptability and stress was not considered, as the necessary criteria for mediation regarding the concept of salvation, as presented by Baron and Kenny in their 1986 study, were not fulfilled in this case.

According to the literature, teenagers develop their sense of self-worth within the family by engaging in effective communication and consistency with their parents, actively contributing to resolving family issues, and adhering to family roles and norms. These factors are crucial for the overall functioning of the family environment [47].

Furthermore, adolescents’ self-esteem is enhanced by parental acceptance and emotional support. Parents’ emotional availability and involvement influence these factors in their relationship with their children, which are crucial aspects of family functioning [41]. Researchers [79] found that dysfunction within the family environment is a strong indicator of low self-esteem in adolescents and children. The study involved a sample of 816 individuals aged 7–16 years.

The literature links self-esteem to the mental health burden of adolescents [25,80], and this study explicitly examines its association with anxiety symptoms [20,51]. The prevailing model in the literature regarding the relationship between self-esteem and anxiety is the vulnerability model. This model suggests that individuals with low self-esteem tend to perceive others as rejecting and to seek constant validation of their worth. Consequently, they are more likely to experience symptoms of anxiety and depression [81,82].

Various studies have examined the mediating role of self-esteem in the relationship between family functioning and stress, in addition to separately assessing the relationship between family dysfunction and self-esteem and the relationship between self-esteem and stress. Researchers [41] found that dysfunction within the family was a contributing factor to low self-esteem in Chinese adolescents. This, in turn, resulted in high levels of social anxiety among them.

Researchers [30] have asserted that the bond between parents and adolescents, particularly during crucial stages of life, is a crucial element in shaping adolescents’ self-esteem. This, in turn, influences the connection of the parent–adolescent interaction and the development of introversion in adolescents. The study [83] illustrates that the significance of the parent–adolescent relationship originates from the initial attachment between the child and the mother during the early years of life. The study found that individuals with an insecure–anxious attachment style were more likely to experience symptoms of anxiety and depression during adolescence. This was attributed to their low self-esteem and dysfunctional self-concepts.

However, research equally corroborates that issues related to introversion in adolescents are prone to result in diminished self-worth and unfavorable perceptions of their family’s performance. Specifically, the bibliography presents the scar model, which explains that adolescents who have anxious and depressive symptoms develop a negative self-image and low self-esteem. This is caused by their feelings of helplessness and distorted self-evaluation, which are influenced by their compromised mental health [82]. As a result, they tend to distance themselves from the family setting, and their interactions with their parents are impacted, which strains the unity of the family context [47].

Furthermore, adolescents with low self-esteem may exhibit symptoms of anxiety as a result of their sense of inadequacy, self-doubt, and negative thinking patterns. Simultaneously, anxiety also contributes to diminished self-esteem, as the adolescent undergoing anxiety likely avoids confronting challenging circumstances. While avoiding the situation may temporarily relieve the symptoms of anxiety, it ultimately prevents them from confronting and effectively dealing with the condition in the long run.

Additionally, it results in a dearth of experiences that would facilitate the acquisition of skills necessary to manage their anxiety symptoms effectively. Consequently, adolescents experience a sense of powerlessness and incompetence, which significantly contributes to their diminished self-worth [52].

Furthermore, there appears to be a reciprocal relationship between family dysfunction and the manifestation of anxiety symptoms in adolescents [84]. To elaborate, it appears that family dysfunction, inadequate parental communication with teenagers, and contentious relationships among family members can make teenagers more susceptible to experiencing stress. Conversely, adolescents experiencing symptoms of anxiety struggle to effectively communicate with their parents and cope with their intense emotional distress. This can result in conflicts between them, the breakdown of communication within the family, and disengagement among family members [85].

The existing evidence has shown that the connections between family dysfunction, self-esteem, and adolescent anxiety symptomatology are intricate and reciprocal, leading to detrimental cycles involving both the adolescent and the family. In order to examine the temporal sequence of these factors, it is necessary to conduct a more significant number of longitudinal studies. Practical support and intervention for the adolescent and the entire family are crucial for managing and cultivating healthy interaction dynamics among family members [30].

Several limitations surfaced during the current investigation, necessitating their acknowledgment and careful consideration in subsequent research endeavors. Initially, the study’s overall sample size is relatively small, particularly for studies that intend to examine potential mediating factors like the present one. This fact leads to the drawing of unreliable conclusions when examining the mediating factor. The lack of representativeness in the sample makes it challenging to apply the findings to the entire population.

In addition, the proportion of e-teen participants between the ages of 15 and 18 is twice as high as those between the ages of 13 and 15. The percentage of teenagers from nuclear families is nearly twice as high as the percentage of teenagers from alternative family structures, such as single-parent, reconstituted, or extended families. As a result, the comparison of various age and family structure categories yields equally uncertain conclusions [86].

Furthermore, the study solely relied on the adolescents’ evaluations of their family’s cohesion and adaptability levels, as well as their anxiety symptoms [87] and self-esteem levels [88,89]. Future studies incorporating parents’ assessments of their family’s functioning may yield more comprehensive results.

Ultimately, the socioeconomic context was thoroughly analyzed by considering the teenagers’ perspective on their families’ socioeconomic status. However, a comprehensive investigation of this factor was not conducted using a diverse range of questions. Furthermore, it is essential to note that the synchronous design of the study precludes making definitive conclusions about the causal relationship between the variables being investigated.

This study established a connection between family cohesion and adaptability and the manifestation of anxious symptoms in adolescents. Furthermore, it explored how adolescent self-esteem plays a role in mediating this relationship. The enrichment of the existing research literature on the above relationship is its positive effect. It also emphasizes the importance of studying the mental well-being of teenagers raised in similar family environments [90,91,92]. Simultaneously, this study emphasizes the safeguarding influence of the self-esteem of these adolescents. It serves as the driving force for developing suitable intervention programs to safeguard the mental equilibrium of this demographic.

Further research could be undertaken on a more extensive cohort of adolescents to extrapolate the findings to the broader population. Simultaneously, longitudinal studies are crucial for documenting the chronological order of the factors. Furthermore, research on comparable subjects could incorporate parents in the data-gathering process and juxtapose their data with the teenagers. Additionally, research on family functionality [93] could explore other significant factors associated with this aspect, including conflicts within the parental partnership, parental psychopathology, and the parental approach to raising adolescents.

## 6. Conclusions

In conclusion, the study confirms the pivotal role of family functioning in adolescents’ mental health and well-being. Family environments characterized by high cohesion, adaptability, and healthy communication are protective against anxiety and are related to increased self-esteem. These positive family dynamics provide a conducive environment for psychological resilience and well-being in adolescents. Conversely, family conflict, dysfunction, and disparities such as socioeconomic challenges significantly contribute to the risk of anxiety symptoms and low self-esteem among adolescents, emphasizing the need for appropriate intervention strategies. The findings highlight the importance of fostering supportive family relationships and addressing family adversity, in addition to reinforcing adolescent self-esteem, to effectively mitigate anxiety symptoms and enhance the overall mental health of adolescents.

## Figures and Tables

**Table 1:** Demographic data of students in total sample and by family type.

		Sample Total	Family Type		
			Extreme	Medium	Moderately Balanced/Balanced
		Ν (%)	Ν (%)	Ν (%)	Ν (%)
Children’s details					
Sex	Boys	81 (48.8)	17 (45.9)	44 (53)	20 (43.5)
	Girls	85 (51.2)	20 (54.1)	39 (47)	26 (56.5)
Age	13–15	50 (30.1)	11 (29.7)	22 (26.5)	17 (37)
	15–18	116 (69.9)	26 (70.3)	61 (73.5)	29 (63)
Class	A’ Gymnasium	1 (0.6)	0 (0)	1 (1.2)	0 (0)
	Second high school	25 (15.1)	4 (10.8)	16 (19.3)	5 (10.9)
	Third high school	24 (14.5)	7 (18.9)	5 (6)	12 (26.1)
	First grade of high school	52 (31.3)	10 (27)	25 (30.1)	17 (37)
	Second grade of high school	27 (16.3)	7 (18.9)	17 (20.5)	3 (6.5)
	Third grade of high school	37 (22.3)	9 (24.3)	19 (22.9)	9 (19.6)
Family details					
Marital status	Together/married	118 (71.1)	24 (64.9)	53 (63.9)	41 (89.1)
	It is in dimension	13 (7.8)	4 (10.8)	7 (8.4)	2 (4.3)
	Divorced	35 (21.1)	9 (24.3)	23 (27.7)	3 (6.5)
Parents alive	Yes	158 (95.2)	3 (8.1)	4 (4.8)	1 (2.2)
	No	8 (4.8)	34 (91.9)	79 (95.2)	45 (97.8)
Family structure	Nuclear	102 (61.4)	22 (59.5)	44 (53)	36 (78.3)
	Extended	14 (8.4)	0 (0)	10 (12)	4 (8.7)
	Single parent	39 (23.5)	12 (32.4)	22 (26.5)	5 (10.9)
	Reconstituted	11 (6.6)	3 (8.1)	7 (8.4)	1 (2.2)
Brothers	No	38 (22.9)	6 (16.2)	19 (22.9)	13 (28.3)
	Yes	128 (77.1)	31 (83.8)	64 (77.1)	33 (71.7)
Occupation of father	State employee	54 (32.5)	10 (27)	30 (36.1)	14 (30.4)
	Private employee	59 (35.5)	13 (35.1)	27 (32.5)	19 (41.3)
	Freelancer	51 (30.7)	14 (37.8)	24 (28.9)	13 (28.3)
	Unemployed	2 (1.2)	0 (0)	2 (2.4)	0 (0)
Occupation of mother	State employee	51 (30.7)	11 (29.7)	28 (33.7)	12 (26.1)
	Private employee	56 (33.7)	15 (40.5)	22 (26.5)	19 (41.3)
	Freelancer	23 (13.9)	3 (8.1)	10 (12)	10 (21.7)
	Unemployed	10 (6)	4 (10.8)	5 (6)	1 (2.2)
	Household	26 (15.7)	4 (10.8)	18 (21.7)	4 (8.7)
Socioeconomic context	Low	38 (22.9)	20 (54.1)	16 (19.3)	2 (4.3)
	Medium	113 (68.1)	16 (43.2)	60 (72.3)	37 (80.4)
	Superior	15 (9)	1 (2.7)	7 (8.4)	7 (15.2)
Father’s educational level	Primary	6 (3.6)	2 (5.4)	2 (2.4)	2 (4.3)
	Secondary	71 (42.8)	21 (56.8)	38 (45.8)	12 (26.0)
	Tertiary	89 (53.6)	14 (37.8)	43 (51.8)	31 (69.6)
Educational level of mother	Primary	2 (1.2)	1 (2.7)	1 (1.2)	0 (0)
	Secondary	58 (34.9)	18 (48.6)	31 (37.3)	9 (19.6)
	Tertiary	106 (63.9)	18 (48.6)	51 (61.4)	37 (80.4)

**Table 2:** Descriptive statistics of FACES, STAI-C, and Rosenberg scales for total sample and by family type.

		Family Type			
	Sample Total	Extreme	Medium	Moderately Balanced/Balanced	
	Median (Range)	Median (Range)	Median (Range)	Median (Range)	χ^2^
Cohesion	34 (28–40)	26 (21–32)	33 (28–38)	42 (38–44)	80.40 (2) ***
Adaptability	25 (22–30)	19 (19–21)	25 (23–27)	31 (27–32)	84.86 (2) ***
Predisposition Anxiety	35 (32–41)	38 (33–41)	36 (32–41)	33 (29–39)	6.91 (2) *
Situational Anxiety	40 (35–45)	41 (38–48)	40 (36–45)	37.5 (33–46)	3.07 (2)
Self-Esteem Scale (Rosenberg)	26 (23–29)	24 (21–29)	26 (23–28)	28 (24–32)	5.86 (2)

* *p* < 0.05, *** *p* < 0.001.

**Table 3:** Descriptive statistics of FACES for total sample and by family type.

			Sample Total	Boys	Girls
		Grade	Ν (%)	Ν (%)	Ν (%)
Cohesion	Detached	1	24 (14.5)	9 (11.1)	15 (17.6)
		2	60 (36.1)	32 (39.5)	28 (32.9)
	Divorced	3	21 (12.7)	13 (16)	8 (9.4)
		4	21 (12.7)	11 (13.6)	10 (11.8)
	Connected	5	17 (10.2)	7 (8.6)	10 (11.8)
		6	15 (9)	7 (8.6)	8 (9.4)
	Very Connected	7	5 (3)	1 (1.2)	4 (4.7)
		8	3 (1.8)	1 (1.2)	2 (2.4)
Adaptability	Rigid	1	4 (2.4)	2 (2.5)	2 (2.4)
		2	21 (12.7)	11 (13.6)	10 (11.8)
	Structured	3	26 (15.7)	14 (17.3)	12 (14.1)
		4	25 (15.1)	15 (18.5)	10 (11.8)
	Flexible	5	24 (14.5)	9 (11.1)	15 (17.6)
		6	20 (12)	9 (11.1)	11 (12.9)
	Very Flexible	7	45 (27.1)	21 (25.9)	24 (28.2)
		8	1 (0.6)	0 (0)	1 (1.2)
Family Type	Extreme		37 (22.3)	17 (21)	20 (23.5)
	Medium		83 (50)	44 (54.3)	39 (45.9)
	Balanced/Moderately Balanced		46 (27.7)	20 (24.7)	26 (30.6)

**Table 4:** Spearman correlation coefficients between cohesion–adaptability, anxiety, and self-esteem scales.

	Cohesion	Adaptability	Predisposition Anxiety	Situational Anxiety
Self-Esteem Scale (Rosenberg)	0.24 **	0.10	−0.42 ***	−0.63 ***
Cohesion	1.00	0.22 **	−0.46 ***	−0.25 **
Adaptability		1.00	0.03	−0.02
Predisposition Anxiety			1.00	0.60 ***

** *p* < 0.01, *** *p* < 0.001.

**Table 5:** Predisposition anxiety and state anxiety scores according to students’ demographic characteristics.

		Predisposition Anxiety		Situational Anxiety	
		Median (Range)		Median (Range)	
			U value		U value
Sex	Boys	33 (31–39)	2553.5 **	37 (33–41)	1780.5 ***
	Girls	38 (33–42)		44 (38–50)	
Age	13–15	33.5 (31–40)	2617.5	42.5 (38–48)	2185 *
	15–18	35.5 (32–41)		39 (34–44.5)	
Marital Status	Together/married	35 (31–41)	2607.5	39 (35–46)	2549.5
	Separated/divorced	36 (32–41)		41 (36.5–45)	
Parents Alive	No	37.5 (33.5–42.5)	497	36.5 (32–41)	438.5
	Yes	35 (31–41)		40 (36–46)	
Nuclear Family Structure	No	35.5 (32–41)	3118.5	39.5 (35–45)	3250.5
	Yes	35 (31–40)		40 (36–46)	
Siblings	No	39 (32–41)	2200.5	40 (38–46)	2144
	Yes	35 (32–41)		40 (35–45)	
			χ^2^		χ^2^
Class	A’ Gymnasium	32 (32–32)	12.38 *	48 (48–48)	8.65
	Second high school	36 (32–40)		43 (39–45)	
	Third high school	33 (30–40.5)		40.5 (36.5–50)	
	First grade of high school	34 (30.5–38)		37 (32.5–44)	
	Second high school	38 (32–43)		40 (37–48)	
	Third high school	40 (33–43)		39 (35–45)	
Father’s Occupation	State employee	35 (31–41)	1.93	40.5 (36–46)	1.83
	Private employee	38 (31–41)		41 (36–46)	
	Freelancer	34 (32–40)		39 (35–45)	
	Unemployed	41.5 (40–43)		40.5 (39–42)	
Mother’s Occupation	State employee	34 (32–40)	4.80	39 (34–46)	6.05
	Private employee	38 (31.5–43)		41 (36–48)	
	Freelancer	35 (32–40)		41 (38–45)	
	Unemployed	34.5 (33–43)		42 (38–45)	
	Household	33.5 (29–41)		37 (33–44)	
Socioeconomic Context	Low	39 (34–42)	6.24 (2) *	42 (39–52)	7.34 (2) *
	Medium	35 (31–40)		39 (35–45)	
	High	34 (30–40)		39 (36–45)	
Father’s Educational Level	Primary	42 (32–44)	1.21 (2)	46.5 (38–51)	2.91 (2)
	Secondary	35 (32–41)		41 (36–45)	
	Tertiary	34 (31–40)		39 (35–45)	
Mother’s Educational Level	Primary	47.5 (44–51)	5.07 (2)	52.5 (52–53)	4.65 (2)
	Secondary	35 (32–42)		40.5 (36–45)	
	Tertiary	35 (31–40)		39 (35–45)	

* *p* < 0.05, ** *p* < 0.01, *** *p* < 0.001.

**Table 6:** Coherence and adaptability dimension scores according to students’ demographic characteristics.

		Cohesion		Adaptability	
		Median (Range)		Median (Range)	
			U value		U value
Sex	Boys	34 (29–38)	3410.5	24 (22–30)	3150
	Girls	34 (28–41)		26 (22–30)	
Age	13–15	33.5 (28–39)	2791.5	26 (22–30)	2738
	15–18	34.5 (29–40.5)		25 (21–30)	
Marital Status	Together/married	37 (31–41)	1616.5 ***	25 (21–30)	2687
	Separated/divorced	30.5 (23.5–34.5)		25 (22–29)	
Parents Alive	No	31 (22–38)	5105.5	22.5 (18.5–24.5)	373 *
	Yes	35 (29–40)		25 (22–30)	
Nuclear Family Structure	No	32 (24.5–37)	2244.5 **	24 (21–28.5)	2808
	Yes	37 (31–41)		26 (22–30)	
Siblings	No	34 (28–39)	2282.5	26 (23–31)	2117
	Yes	34 (29–40.5)		25 (21.5–30)	
			χ^2^		χ^2^
Class	A’ Gymnasium	44 (44–44)	11.19 (5) *	22 (22–22)	1.11 (5)
	Second high school	32 (26–36)		25 (23–29)	
	Third high school	37 (31.5–42.5)		26 (22–30.5)	
	First grade of high school	36 (31–41)		25 (22–29.5)	
	Second high school	33 (24–40)		25 (20–31)	
	Third high school	33 (29–40)		25 (21–30)	
Father’s Occupation	State employee	34 (30–41)	2.38 (3)	25 (22–31)	0.21 (3)
	Private employee	35 (28–40)		25 (23–30)	
	Freelance	34 (28–40)		25 (20–30)	
	Unemployed	26.5 (22–31)		26 (26–26)	
Mother’s Occupation	State employee	34 (29–40)	2.29 (4)	25 (21–30)	4.75 (4)
	Private employee	35.5 (28.5–41)		26 (22–31)	
	Freelance	36 (31–42)		26 (23–30)	
	Unemployed	33 (26–38)		22 (19–25)	
	Household	35.5 (27–40)		23.5 (20–30)	
Social–Economic Context	Low	26 (21–33)	27.62 (2) ***	22 (19–25)	20.53 (2) ***
	Medium	36 (31–41)		26 (23–30)	
	Superior	36 (30–44)		30 (24–31)	
Father’s Educational Level	Primary	36 (21–44)	2.17 (2)	25 (22–32)	6.38 (2) *
	Secondary	33 (28–39)		24 (21–27)	
	Tertiary	36 (29–41)		27 (22–31)	
Mother’s Educational Level	Primary	25.5 (21–30)	3.80 (2)	22.5 (12–33)	8.39 (2) *
	Secondary	33 (27–39)		23 (20–26)	
	Tertiary	35 (29–41)		26 (23–30)	

* *p* < 0.05, ** *p* < 0.01, *** *p* < 0.001.

**Table 7:** Results of hierarchical linear regression with predisposition anxiety dimension as dependent variable.

Model	Independent Variables	b	SE	b‡	t
1: F(2.163) = 22.78, *p* < 0.001, R^2^ = 0.21	Sex (Girls vs. Boys)	0.031	0.011	0.20	2.88 **
	Cohesion	−0.004	0.001	−0.42	−6.06 ***
2: F(3.162) = 21.64, *p* < 0.001, R^2^ = 0.27 R^2^ = 0.06	Sex (Girls vs. Boys)	0.018	0.011	0.12	1.65
	Cohesion	−0.004	0.001	−0.36	−5.24 ***
	Self-Esteem Scale (Rosenberg)	−0.005	0.001	−0.28	−3.92 ***

** *p* < 0.01, *** *p* < 0.001.

**Table 8:** Collinearity statistics of predisposition anxiety dimension as dependent variable.

Coefficients ^a^							
Model	Unstandardized Coefficients		Standardized Coefficients	t	Sig.	Collinearity Statistics	
	B	Std. Error	Beta			Tolerance	VIF
(Constant)	1.326	0.010	0.029	3.65	<0.001	0.967	1.139
Sex (Girls vs. Boys)	0.031	0.011	0.031	2.88	<0.001	0.917	1.234
Cohesion	−0.004	0.001	−0.004	−6.06	<0.001	0.865	1.240
Self-Esteem Scale (Rosenberg)	−0.005	0.001	−0.005	−3.92	<0.001	0.889	1.309

^a^ Dependent variable: predisposition anxiety dimension.

**Table 9:** Results of hierarchical linear regression with state anxiety dimension as dependent variable.

Model	Independent Variables	B	SE	b‡	t
1: F(3.162) = 19.45, *p* < 0.001, R2 = 0.25	Sex (Girls vs. Boys)	0.069	0.011	0.42	6.23 ***
	Age (15–18 vs. 13–15)	−0.036	0.012	−0.21	−3.04 **
	Cohesion	−0.002	0.001	−0.23	−3.40 **
2: F(4.161) = 37.09, *p* < 0.001, R2 = 0.47 R^2^ = 0.22	Sex (Girls vs. Boys)	0.044	0.010	0.27	4.45 ***
	Age (15–18 vs. 13–15)	−0.029	0.010	−0.16	−2.83 **
	Cohesion	−0.001	0.001	−0.12	−2.03 *
	Self-Esteem Scale (Rosenberg)	−0.008	0.001	−0.50	−8.15 ***

* *p* < 0.05, ** *p* < 0.01, *** *p* < 0.001.

**Table 10:** Collinearity statistics of state anxiety dimension as dependent variable.

Coefficients ^a^							
Model	Unstandardized Coefficients		Standardized Coefficients	t	Sig.	Collinearity Statistics	
	B	Std. Error	Beta			Tolerance	VIF
(Constant)	2.525	0.410	0.071	6159	<0.001	0.989	1.089
Sex (Girls vs. Boys)	0.42	0.011	0.069	6.23	<0.001	0.997	1.003
Age (15–18 vs. 13–15)	−0.21	0.012	−0.036	−3.04	<0.001	0.863	1.159
Cohesion	−0.23	0.001	−0.002	−3.40	<0.001	0.855	1.170
Self-Esteem Scale (Rosenberg)	−0.50	0.001	−0.008	−8.15	<0.001	0.828	1.207

^a^ Dependent variable: state anxiety dimension.

## Data Availability

The data presented in this study are available upon request from the corresponding author. The data are not publicly available due to privacy and ethical concerns.
